# Randomized control trial: Comparison of Triple Therapy plus Probiotic Yogurt vs. Standard Triple Therapy on Helicobacter Pylori Eradication

**Published:** 2012-10-30

**Authors:** V Mirzaee, O Rezahosseini

**Affiliations:** 1 Department of Gastroenterology, Rafsanjan University of Medical Sciences, Rafsanjan, Iran; 2 Medical Student of Rafsanjan University of Medical Sciences, Rafsanjan, Iran

**Keywords:** Helicobacter pylori, probiotic yogurt, triple therapy, eradication

## Abstract

**Background:**

Treatment of Helicobacter pylori (H. pylori) as a major cause of gastric diseases is of utmost concern.We aimed to assess efficacy of triple therapy (amoxicillin, clarithromycin and pantoprazole) plus probiotic yogurt (PY) on eradication of H.pylori.

**Methods:**

Total 102 H. pylori positive patients were divided to 3 groups equally and randomly. For treatment of each group Amoxicillin, clarithromycin and pantoprazole were used. Group A had additional PY, Group B ordinary low fat yogurt and Group C just had triple therapy in their regimen as well. These groups were compared regarding treatment success.

**Results:**

Total number of 88 patients finished the treatment course. The most common experienced side effects were dysgeusia in groups A and B (25.8% and 32.3%, respectively), and dysgeusia with diarrhea and abdominal pain (30.8%) in group C. Eradication rate was, respectively, 61.3%, 64.5% and 71.3% in group A, B and C of which difference was not statistically significant (P> 0.05). However, the difference between 3 groups in regard to education level was statistically significant (P=0.005).

**Conclusion:**

PY enriched triple therapy has decreased side effects of antibiotics consumption; however, this has no impact on eradication of Hpylori. PY and triple therapy can be used concomitantly to increase the patient tolerance.

## Introduction

(1)*Helicobacter pylori* (Hpylori) is a very common infection worldwide which can cause variety of upper gastrointestinal (GI) disorders such as peptic ulcer disease, dyspepsia, atrophic gastritis, gastric hyperplastic polyps, gastric mucosaassociated lymphoid tissue (MALT) lymphoma and gastric carcinoma (1-3). It is believed that about 50% of world population is contaminated with this bacterium which tends to be more in developing countries (4). Eradication of Hpylori is based on regimens of triple- and quadruple therapy including proton pump inhibitor (PPI) + clarithromycin + amoxicillin/metronidazole or bismuth + PPI + tetracycline + metronidazole which are still valid and can be used as first and second lines of treatment, respectively (5). Noticeably, however, increasing pattern of antibiotic resistance is observed within the regimens and alternative regimes have been proposed (e.g. levofloxacin/rifabutin + PPI + amoxicillin) which should be validated before their worldwide consumption (5, 6).

Apart from that, the curative impact of probiotic yogurt (PY) on acute non- inflammatory gastroenteritis, atopic eczema, post-operative pouchitis and inflammatory bowel disease has been demonstrated (7-10). Moreover, it has been suggested that PY can increase patient compliance of Hpylori eradication agents through decreasing the adverse effects (11, 12).

According to anti-inflammatory effect of PY, increasing bacterial resistance to antibiotics specially clarithromycin and negative impact of drug adverse effects on patient compliance (13), for the first time we aimed to perform this study to compare the eradicative influence of triple therapy (PPI + clarithromycin + amoxicillin) in groups of patients who receive probiotic and nonprobiotic yogurt.

## Materials and Methods

### Study design

From Feb 2009 to Sep 2010 all patients of upper GI tract complaints (age range 18-85 years) who had came/been referred to the Gastroenterology Clinic at Ali-ibn-Abi Taleb Hospital affiliated to Rafsanjan University of Medical Sciences (Rafsanjan, Iran) were considered for the study. According to the equation 1, 34 patients in each of the below mentioned group (17 females and 17 males) are needed. Therefore urea breath test (UBT) with carbon-13 (13C) for all cases suspicious for Hpylori infection was performed and patients with positive results, who did not match the exclusion criteria, were registered so that they would be allocated in the 3 treatment groups. The inclusion criteria were, age range 18-85, positive UBT and willingness to participate in study.

**equation 1:**
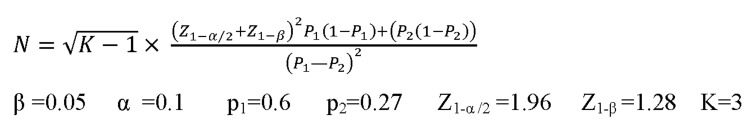
Calculation of sample size

The exclusion criteria included past history of complete or incomplete Hpylori eradication, suspicious or proven ongoing malignancy, history of gastric surgery, pregnant or breast-feeding women, intake of antibiotics, PPI or bismuth within past 2 months and present history of hypersensitivity reaction to penicillin or clarithromycin.

This cohort was approved by the Ethical Board of the Rafsanjan Medical University and all patients signed the consent form of study enrollment.

The study registered to Iranian Registry of Clinical Trials in: IRCT138904011061N9.

### Data gathering

Questionnaires were used to record demographics and medical history/exam. Result of treatment and follow-up was recorded as well.

### Treatment and follow-up

Using table of random numbers, patients were allocated equally to one of the following treatment categories: A) pantoprazole 40 mg once daily, amoxicillin 1 gr twice daily, clarithromycin 500 mg twice daily and PY (1.5% fat) 150mgtwice daily. B) pantoprazole 40 mg once daily, amoxicillin 1 gr twice daily, clarithromycin 500 mg twice daily and non-PY (1.5% fat and from the same dairy) 150mg twice daily. C) pantoprazole 40 mg once daily, amoxicillin 1 gr twice daily and clarithromycin 500 mg twice daily. All drugs were administered orally for 7 days and patients were asked not to have dairy products in their food regimen unless as it was ordered.

After treatment commenced, all patients have been assessed for compliance, adverse reaction and any problem in GI tract and, in the end, follow-up UBT was performed 4 weeks after treatment ended to evaluate if the eradication strategies were successful.

### Urea breath test (UBT)

UBT was done while all patients had been requested not to eat or have injection four hours prior to the test. First sample was obtained before ingestion of oral radionuclide. Second sample was obtained 30 minutes after when the 13C-containing urea tablet with one glass orange juice was ingested. Isotope ratio mass spectrometry (IRMS) was used for sampling.

Patients who have not properly followed the instructions of drug and/or yogurt use due to any reason and who have not participated in UBT were all disregarded.

### Statistical analysis

The SPSS 17 was used for statistical analysis. The positive or negative result of UBT was compared by ANOVA test among the 3 groups. Moreover, a comparison between each demographic character within our groups and the result of UBT was performed by t-test. The level of statistical significance and the confidence interval (CI) were established as p < 0.05 and 95%, respectively.

## Results

### Demographic data

In total, 102 patients with proven Hpylori infection were selected for the study from which 34 individuals (17 females and 17 males) were allocated into 3 groups using table of random numbers. Three individuals in group A (1 female, 2males), 3 individuals in group B (3 females) and 8 individuals (2 females, 6 males) in group C left the study.

Mean and standard deviation (SD) of age, body mass index (BMI), delta over baseline (DOB) levels of before and after treatment (DOBB and DOBA) are summarized in table 1.

**Table 1:** Mean of age, BMI and DOB in each group

Mean	Age	BMI	DOB before treatment	DOB after treatment
**Group A**	31.3±12.6	23.2±3.1	21.3±13.2	6.9±9.5
**Group B**	35.2±8.0	24.2±4.2	20.6±13.3	4.8±6.4
**Group C**	39.1±14.5	22.7±3.1	14.3±11.0	2.5±5.5

BMI: body mass index, DOB:Delta over baseline

**Figure 1:**
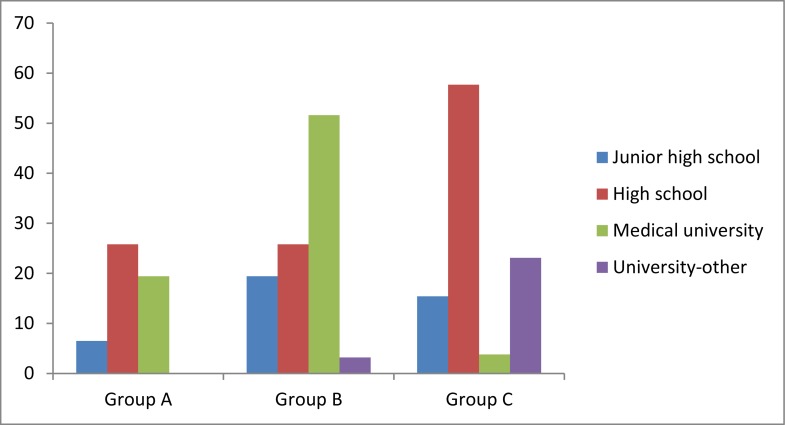
Frequency of each of the four education levels within our groups (%)

**Figure 2:**
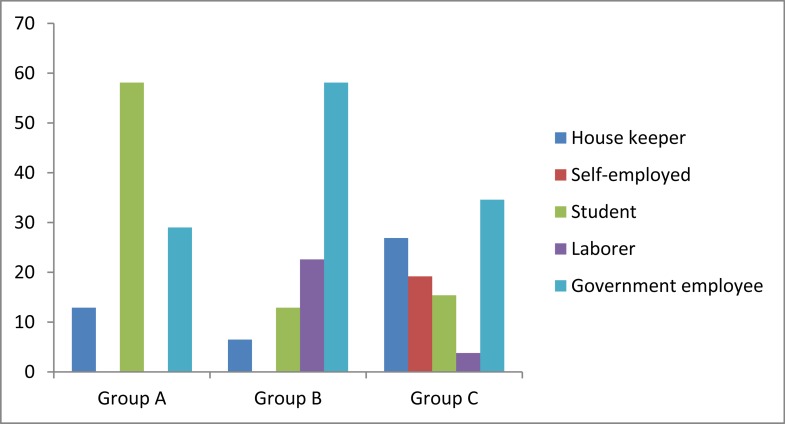
Frequency of job categories within our groups (%)

Moreover, education level and job category of patients are demonstrated in figures 1 and 2, respectively.

### Treatment outcome

**Figure 3:**
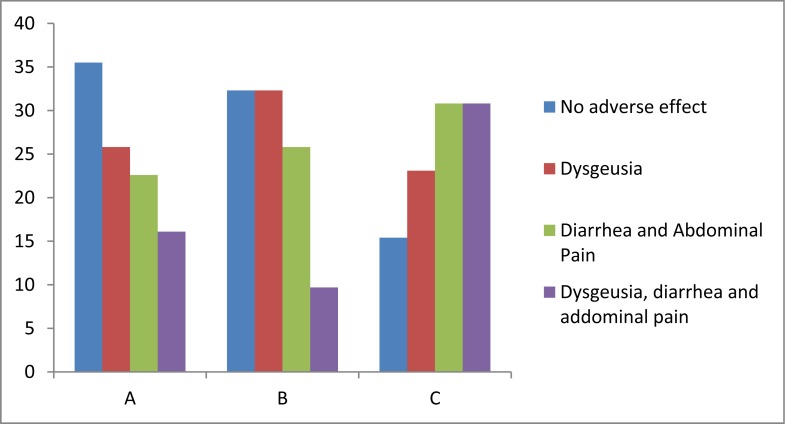
Frequency of adverse effects observed within our groups (%)

Figure 3 demonstrates adverse effects occurred within our population. Dysgeusia was the most frequent adverse effect in patients who had yogurt in their treatment. In group C, however, dysgeusia, diarrhea and abdominal pain were most noticed.

In groups A, B and C, in 19 (61.3%), 20 (64.5%) and 19 (73.1%) patients eradication regimen were successful, respectively. The achieved eradication rate was the highest in group C, however, this was not statistically significant.

Successful (*helicobacter pylori* eradication + (HPE+)) and unsuccessful (*helicobacter pylori* eradication − (HPE−)) eradication categories were compared in regard to each of age, sex, education level, job category, BMI, DOBB, DOBA and adverse reaction parameters of which results follow (table 2):

**Table 2:** Comparison of eradication success in each group regarding age, sex, education level, job category, BMI, DOB and adverse reaction

		Group A		Group B		Group C	
	HPE	Mean	p-value	Mean	p-value	Mean	p-value
**Age**	+	31.4±12.1	0.985	34.2±8.4	0.318	37.2±14.6	0.285
	−	31.3±13.9		37.2±7.3		44.2±14.3	
**Sex (years)**	+	1.4±0.5	0.195	1.4±0.5	0.453	1.5±0.5	0.974
	−	1.6±0.5		1.5±0.5		1.5±0.5	
**Education level**	+	3.3±1	0.132	3.1±1.0	0.176	2.3±0.7	0.061
	−	2.7±0.9		2.4±1.5		1.7±0.4	
**Job**	+	3.3±1	0.971	4.1±1.3	0.480	3.1±1.6	0.607
	−	3.3±1.4		4.4±0.5		2.7±1.7	
**BMI**	+	22.9±3.1	0.599	24.7±3.8	0.399	22.1±3.1	0.117
	−	23.6±3.2		23.3±5.1		24.3±2.4	
**DOB before treatment**	+	14.7±8.4	<0.001	16.0±11.3	0.007	12.0±9.6	0.086
	−	31.7±13.1		29.0±13.1		20.5±13.5	
**DOB after treatment**	+	1.0±2.0	<0.001	1.5±2.9	<0.001	0.8±3.0	0.005
	−	16.4±9.2		10.4±6.9		7.8±8.0	
**Adverse reactions**	+	1.0±1.1	0.382	1.1±1.0	0.830	1.6±1.1	0.289
	−	1.4±1.0		1.1±0.9		2.1±0.9	

Group A: Between HPE+ and HPE− patients, DOBB and DOBA levels, the mean values were 14.7±8.4 and 31.7±13.1 (p<0.001), 1.0±2.0 and 16.4 ±9.2 (p<0.001), for DOBB and DOBA, respectively. No other parameter was found to be significantly different between HPE+ and HPE− patients.

Group B: as well as previous group, DOBB and DOBA levels were detected to have significant difference between HPE+ and HPE− patients. The mean values were 16.0±11.3 and 29.0±13.1 (p=0.007) , 1.5±2.9 and 10.4±6.9 (p= 0.001) in DOBB and DOBA, respectively. None of the other parameters were found to bring significant difference between HPE+ and HPE− patients.

Group C: In HPE+ sub group, DOBA was lesser in compare to HPE− subgroup (means: 0.8 ±3.0 and 7.4 ±8.0, respectively and p=0.005). However, this difference was not significant in DOBB and other parameters.

In addition to the above mentioned results, comparison between the three groups in each of HPE+ and HPE− subgroups was done (table 3). As it has been shown, education level of HPE+ population (p=0.005) and job category of HPE− population (p=0.020) were difference between the 3 groups. Hence, we have reached the fact that the mentioned difference in education level is resulted from the difference between groups A and C and groups B and C (p=0.006 and 0.034, respectively), however, no difference was found between groups A and B (p=0.761). Additionally, the achieved difference in jobs of HPE− patient groups is limited to the difference between groups B and C (p=0.021) only and this parameter had no difference between groups A and B, A and C, (p=0.102 and 0.563, respectively). Statistically significant difference between the study groups was found regarding none of the remaining parameters.

**Table 3:** Comparison of study groups with each other regarding age, sex, education level, job category, BMI, DOB and adverse reaction

		HPE +		HPE −	
	Group	Mean	p-value	Mean	p-value
**Age**	**A**	**31.4±12.1**	**0.328**	**31.3±13.9**	**0.092**
	**B**	**34.2±8.4**		**37.2±7.3**	
	**C**	**37.2±14.6**		**44.2±14.3**	
**Sex**	**A**	**1.4±0.5**	**0.492**	**1.6±0.5**	**0.841**
	**B**	**1.4±0.5**		**1.5±0.5**	
	**C**	**1.5±0.5**		**1.5±0.5**	
**Education level**	**A**	**3.3±1**	**0.005**	**2.7±0.9**	**0.172**
	**B**	**3.1±1.0**		**2.4±1.5**	
	**C**	**2.3±0.7**		**1.7±0.4**	
**Job**	**A**	**3.3±1.2**	**0.062**	**3.3±1.4**	**0.020**
	**B**	**4.1±1.3**		**4.4±0.5**	
	**C**	**3.1±1.6**		**2.7±1.7**	
**BMI**	**A**	**22.9±3.1**	**0.060**	**23.6±3.2**	**0.877**
	**B**	**24.7±3.8**		**23.3±5.1**	
	**C**	**22.1±3.1**		**24.3±2.4**	
**DOB before treatment**	**A**	**14.7±8.4**	**0.453**	**31.7±13.1**	**0.217**
	**B**	**16.0±11.3**		**29.0±13.1**	
	**C**	**12.0±9.6**		**20.5±13.5**	
**DOB after treatment**	**A**	**1.0±2.0**	**0.656**	**16.4±9.2**	**0.066**
	**B**	**1.5±2.9**		**10.4±6.9**	
	**C**	**0.8±3.0**		**7.8±8.0**	
**adverse reactions**	**A**	**1.0±1.1**	**0.198**	**1.4±1.0**	**0.153**
	**B**	**1.1±1.0**		**1.1±0.9**	
	**C**	**1.6±1.1**		**2.1±0.9**	

## Discussion

According to what we has been found, in contrast and despite the detected different eradication rates between the 3 groups which represented more success by group C (success rates: A: 61.3%, B: 64.5% and C: 73.1%), none of these differences were significant and apparently triple therapy with PY is unlikely to have a significant impact on HPE than other regimens used in this study and, therefore, cannot be effectively yielded by PY-enriched regimens. Moreover, Kim et al has shown that concomitant administration of probiotics and triple therapy can increase the eradication rate; however, they found this approach to have no effect on lowering the adverse effects (14).

Nonetheless, patients who have not received yogurt -of any type- (group C) have more adverse reaction and they suffered most from abdominal pain and dysgeusia. Although patients who had PY or non-PY in their treatment regimen experienced less adverse reactions and only dysgeusia without abdominal pain was the prominent one, this differences were not significant for both HPE+ and HPE− patients. This, however, is similar to the reports of other investigators who have suggested the use of probiotic bacteria to increase the compliance and intend to treat of patients (14, 15). The literature, however, lacks any study in which PY and triple therapy are used to eradicate Hpylori.

Apart from that, the levels of DOBB and DOBA were compared between HPE+ and HPE− and we realized that HPE+ patients have significantly lower amount of DOB. Mean DOBB levels of HPE− patients in groups A, B and C were 17.0 %, 13 % and 8.5 % lower than of HPE+ patients. Similarly, mean DOBA levels of HPE− individuals were significantly lower than of HPE+ individuals for 15.4, 8.9 and 7 % in A, B and C groups, respectively. This may suggest the correlation between level of DOB and severity of Hpylori infection which may demand more effective treatment. Nevertheless, no study has evaluated this correlation so far.

In parallel, parameters such as age, sex, education level, job, BMI, DOBB, DOBA and presented adverse reactions were analyzed to evaluate if each of them differs among the 3 groups while HPE+ and HPE− patients are divided. We found that age, sex, BMI, DOB and adverse reactions of the treatment do not differ significantly between the 3 groups within both HPE+ and HPE− individuals. In addition, education level and job of our population were not significantly different in HPE− and HPE+, respectively. This represents the normal distribution pattern of total population among the groups. However, HPE+ and HPE− patients were significantly different regarding education level and job, respectively. This is occurred due to the fact that our patients have not been matched unless for sex. Besides, the role of race (16), should not be neglected which can confound the results specially studies with great sample size such as national, international or referral centers’ studies.

Adverse effects of antibiotics and consequently low patients’ tolerance especially in group C were two limitations in present study that were unavoidable. Some patient did not cooperate to finish the period of treatment, and we persuaded them to continue by phone call follow up. We conclude that PY cannot affect the result of HPE however, can be used to decrease the adverse effects (e.g. abdominal pain) which can indirectly maximize the success rate of eradication.
